# Plasma and Cellular Forms of Fibronectin as Prognostic Markers in Sepsis

**DOI:** 10.1155/2020/8364247

**Published:** 2020-08-01

**Authors:** Anna Lemańska-Perek, Dorota Krzyżanowska-Gołąb, Tomasz Skalec, Barbara Adamik

**Affiliations:** ^1^Department of Chemistry and Immunochemistry, Wroclaw Medical University, M. Skłodowskiej-Curie 48/50, 50-369 Wrocław, Poland; ^2^Department of Anesthesiology and Intensive Therapy, Wroclaw Medical University, Borowska 213, 50-556 Wroclaw, Poland

## Abstract

**Background:**

There is a pressing need for specific prognostic markers that could be used to monitor the severity of sepsis. The aims of our study were to investigate changes in the expression of different molecular forms of fibronectin in sepsis and to assess their relationship to the clinical severity and mortality of patients. *Material and Methods*. Forms of fibronectin: plasma (pFN), cellular (EDA-FN), FN-fibrin complexes, and fibronectin fragments were analyzed in 71 sepsis patients (survivors and nonsurvivors) and in the control by ELISA and immunoblotting.

**Results:**

The baseline pFN concentration of patients with sepsis was significantly lower than in the control (133.0 mg/L vs. 231.2 mg/L) (*P* < 0.001), and in nonsurvivors, it was lower than in survivors (106.0 mg/L vs. 152.8 mg/L) (*P* = 0.004). The baseline EDA-FN was significantly elevated in both sepsis groups (survivors: 6.7 mg/L; nonsurvivors: 9.4 mg/L) compared to the control (1.4 mg/L) (*P* < 0.001). It should be noted that among patients with more severe sepsis, the EDA-FN level was higher in nonsurvivors than in survivors. Furthermore, molecular FN-fibrin complexes as well as FN fragments occurred much more frequently in nonsurvivors than in survivors.

**Conclusion:**

The study showed that in sepsis, changes in plasmatic and cellular form of fibronectin were associated with the severity of sepsis and may be useful predictors of outcome.

## 1. Introduction

Sepsis is life-threatening organ dysfunction resulting from a dysregulated host response to infection [1]. The diagnosis of sepsis is complicated by the highly variable and nonspecific nature of the signs and symptoms, and mortality in sepsis is high and ranges from 19.3 to 47.2% [2]. In sepsis, the body's immune system responds abnormally to infection by attacking its own tissues and organs, leading to organ failure. Excessive activation of systemic inflammation causes cellular dysfunction, coagulopathies, endothelial dysfunction, and cardiovascular failure. As a consequence, septic shock and multiorgan dysfunction syndrome develop [3]. Our previous studies showed that coagulation abnormalities are present in a majority of patients with sepsis and are associated with a significantly higher mortality rate [4]. There is a pressing need for early, sensitive, and specific biomarkers that would indicate the presence of sepsis and could be used to monitor the severity of sepsis during treatment in the intensive care unit.

Fibronectin (FN) is a high-molecular weight glycoprotein involved in many processes, including cell adhesion, proliferation, embryonic development, and matrix remodelling [5]. There are two defined types of FN: soluble plasma fibronectin (pFN), which is produced by hepatocytes and circulates in soluble form in the blood, and insoluble cellular fibronectin (cFN), which accumulates in tissues as a component of the extracellular matrix [5, 6]. Because of alternating splicing of the FN gene, the cellular form of FN contains extra domains A (isoform EDA) and B (isoform EDB), which are absent or appear in trace amounts in the blood of a healthy human [7]. There is much evidence pointing to the role of FN in the development of various diseases, e.g., in atherosclerosis [8, 9], lung or liver fibrosis [10, 11], diabetes [12], and cancer [13]. It is also known that FN significantly accelerates healing and reduces areas of inflammation, and it is a significant component of a blood clot [5, 14]. Additionally, FN plays an essential role in the host response to infection, being involved in maintaining vascular integrity and wound healing and triggering blood clotting processes [15]. It mediates important interactions of phagocytes throughout the inflammatory process, and by the formation of a three-component bridge, FN contributes to bacterial colonization of endothelial and epithelial cells [15, 16]. The importance of fibronectin in sepsis remains unclear, and very few results have been published so far. According to previous research, the concentration of plasma FN was significantly reduced in patients with sepsis compared to values measured in healthy volunteers [17], and pFN was indicated as an early sepsis marker. Additionally, lower plasma FN levels were observed in cases of fungal sepsis than in cases of bacterial sepsis [18].

The present study was undertaken to investigate changes in the expression of different forms of fibronectin (plasma fibronectin, cellular isoform EDA-fibronectin, supramolecular FN-fibrin complexes, and blood fibronectin fragments) in sepsis and to establish their relationship with the severity of sepsis and mortality of sepsis patients.

## 2. Materials and Method

This observational, prospective study was conducted at the Department of Chemistry and Immunochemistry (the analysis of fibronectin and its isoforms) and at the Department of Anaesthesiology and Intensive Therapy (blood sample collection, clinical data base) of Wroclaw Medical University. The study protocol complies with the 1975 Declaration of Helsinki, as revised in 1983. The study was approved by the Bioethical Committee of Wroclaw Medical University (no. 637/2014), and informed consent was obtained from the patients.

### 2.1. Patients

#### 2.1.1. Inclusion Criteria

The inclusion criteria are as follows: age ≥18 years old, documented or suspected infection, organ dysfunction identified as an acute change in the total SOFA score ≥2 points due to the infection, and a diagnosis of sepsis or septic shock, according to the Sepsis-3 definition, on admission to the intensive care unit [1].

#### 2.1.2. Exclusion Criteria

The exclusion criteria are as follows: pregnancy, terminal illness with no chance for meaningful recovery, and expected ICU length of stay of 24 hours or less.

All patients admitted to the ICU from January 2017 to December 2017 who met the inclusion criteria were included in the study. The severity of sepsis was determined using the APACHE II (Acute Physiology and Chronic Health Evaluation II) score on admission to the ICU. The score is made up of 12 physiological variables and 2 disease-related variables, and it is routinely used as a prediction tool for ICU patients [19]. The extent of organ dysfunction/failure was assessed with the SOFA (Sequential Organ Failure Assessment) score on admission to the ICU and on the 3^rd^, 5^th^, and 8^th^ day of treatment. The score is routinely used in the ICU for monitoring the severity of sepsis based on the status of the following systems: respiratory (PaO_2_/FiO_2_ index), cardiovascular (mean arterial pressure and the dose of vasopressors), hepatic (bilirubin level), coagulation (platelets level), renal (creatinine level/urine output), and neurological (Glasgow coma scale) [20]. All patients in the study received standard treatment according to the Surviving Sepsis Campaign Guidelines [21]. The incidence of disseminated intravascular coagulation (DIC) induced by sepsis was diagnosed according to the International Society on Thrombosis and Haemostasis (ISTH) score [22]. The results of routinely measured parameters were also recorded and analyzed in association with the fibronectin value. The number of ICU-free days was calculated for sepsis patients as 28 minus the number of days in ICU [23, 24].

According to their survival status, all sepsis patients were classified into one of two groups: survivors and nonsurvivors. The control group consisted of healthy volunteers, and blood samples in this group were obtained from the Wroclaw Medical University Biobank.

### 2.2. Blood Sampling

Blood samples (2.7 ml), anticoagulated with 3.2% sodium citrate, were collected from patients diagnosed with sepsis or septic shock on the day of admission to the ICU and on the 3^rd^, 5^th^, and 8^th^ day of treatment. Plasma was immediately separated from blood cells by centrifugation at 2000×g for 10 min, aliquoted, and stored at −70°C for further analysis.

### 2.3. FN Concentration

Plasma FN concentrations were determined by an enzyme-linked immunosorbent assay (ELISA) using a well-defined domain-specific monoclonal antibody directed to cell-binding domain of FN (FN30-8; M010 TaKaRa Shuzo Co. Ltd., Shiga, Japan) as described earlier [25]. The monoclonal antibodies anti-FN were used as a coating agent. The amount of FN bound by the monoclonal antibody was quantified by rabbit anti-FN polyclonal antibodies (Sigma Chemical Co, St. Louis, MO, USA) and peroxidase-conjugated goat anti-rabbit immunoglobulins (Sigma Chemical Co, St. Louis, MO, USA) as the secondary antibodies.

### 2.4. EDA-FN Concentration

EDA-FN concentrations were determined by ELISA using a domain-specific primary antibody (S-FN5, clone IST-9, Sirius Biotech S.r.l., Genoa, Italy) and a biotinylated secondary antibody (715-066-151, Jackson ImmunoResearch, Baltimore, USA). Detection of the EDA domain in plasma was based on the method described by Ziffels et al. [26]. A cellular fibronectin from human foreskin fibroblasts (Sigma, St. Louis, MO, USA, from 1.5 to 50.0 ng/well) was used as a standard.

### 2.5. Western Immunoblotting

Plasma samples containing 300 ng of FN were subjected to SDS (10%)-polyacrylamide gel electrophoresis under reducing conditions as described earlier [27]. The nitrocellulose blots were incubated with rabbit anti-FN antibody (Sigma, diluted 1 : 5000). Anti-rabbit IgG HRP-conjugated (Sigma, dilution 1: 5000) was used as a secondary antibody. The molecular weights of the FN bands were estimated using Triple Color Protein standard III (Serva electrophoresis GmbH, Heidelberg, Germany).

### 2.6. SDS-Agarose FN Immunoblotting

Supramolecular forms of plasma FN were revealed by SDS-agarose immunoblotting as described previously [28]. The plasma sample containing 300 ng of FN was loaded on 1.5% agarose (Standard Low-M_r_, Bio-Rad Laboratories, Hercules, CA, USA), then separated in the Sub-Cell GT System (Bio-Rad Laboratories, Hercules, CA, USA), and transferred to a nitrocellulose membrane (Serva Electrophoresis GmbH, Heidelberg, Germany). The membrane was subjected to immunoblotting with anti-FN monoclonal antibody (FN 30-8; TaKaRa Shuzo Co. Ltd., Japan) and rabbit anti-mouse immunoglobulins conjugated to horseradish peroxidase (Sigma, St. Louis, MO, USA). The relative amounts of the FN molecular forms were expressed as the percentage of the total number of pixels in a lane.

### 2.7. Statistics

All analysis was performed with Statistica 13 software (StatSoft, Inc. Tulsa, USA). The distribution was not normal based on the Shapiro-Wilk test. Therefore, statistical analysis was performed using nonparametric tests. Continuous variables were presented as the median and quartiles (25^th^ and 75^th^). The comparison of continuous variables across three groups (nonsurvivors, survivors, control) was performed using the Kruskal-Wallis test, and a post hoc was used to compare subgroups. The comparison of continuous variables between two independent groups (nonsurvivors vs. survivors) was performed using the Mann-Whitney *U* test. The Friedman repeated-measures ANOVA on ranks with the post hoc test was applied to repeated measurements. Univariate multiple regression and multivariate analysis were performed using logistic regression to evaluate the association between APACHE II and SOFA scores, PCT, fibronectin and mortality; the results were reported as the odds ratio (OR) and 95% confidence intervals (CI). Categorical variables were analyzed using the chi-squared test, and contingency tables were used to analyze the frequency distribution of categorical variables. *P* values less than 0.05 were regarded as significant.

## 3. Results

**Figure 1:**
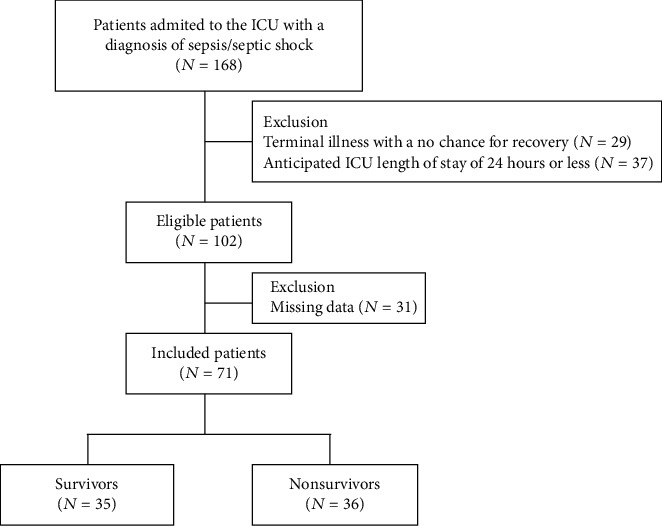
Flow chart of the study.

The baseline characteristics of the study groups are presented in Table 1. Seventy-one patients met the inclusion criteria and were enrolled into the study (Figure 1). According to the survival status, all sepsis patients were classified into one of two groups: survivors (*n* = 35, 17 females and 18 males; median age 68 years) and nonsurvivors (*n* = 36, 19 females and 17 males, median age 73 years). The control group consisted of healthy volunteers (*n* = 17, 4 females and 13 males, median age 35 years). The severity of sepsis on admission to the ICU was determined based on the APACHE II and SOFA scores; a higher score indicated that the patient was in a worse condition. Survivors had significantly lower baseline scores than nonsurvivors (APACHE II: 19.0 vs. 26.0, *P* < 0.001; SOFA: 9.0 vs. 13.0, *P* < 0.001, respectively).

The activation of the systemic inflammatory reaction in sepsis was monitored with a C-reactive protein (CRP), procalcitonin (PCT), and white blood cell count (WBC). Baseline values of CRP and WBC were similar in survivors and nonsurvivors (CRP: 169.0 mg/L vs. 178.6 mg/L and WBC: 15.0 × 10^3^/*μ*L vs. 15.1 × 10^3^/*μ*L, respectively). The PCT level was significantly lower in survivors (2.9 ng/mL) than in nonsurvivors (13.1 ng/mL) (*P* < 0.009). Changes in the indices of inflammatory response over time are presented in a supplement (Table S1). In univariate logistic regression, the initial pFN, APACHE II, SOFA, and PCT were independent predictors of mortality (*P* < 0.05), while the initial EDA was not significant as a mortality predictor. In multivariate logistic regression analysis, only the initial pFN and APACHE II score were independent predictors of mortality (Table 2).

### 3.1. Routine Indices of Coagulation

Survivors were characterised by a platelet count within a normal range, slightly elevated INR at baseline and within a normal range in subsequent days, and elevated D-dimers during the entire observation time. In nonsurvivors, the mean level of platelets was within a normal range at baseline and decreased in subsequent days, and INR and D-dimers were elevated during the entire observation time. Statistically significant changes over time were recorded for standard coagulation parameters: the Friedman ANOVA test followed by a post hoc test indicated a significant decrease in platelet count on day 5 in both study groups, and INR decreased significantly on days 5 and 8 in both study groups (Table 3). However, these statistically significant changes appear to be clinically significant only for changes in platelet count in nonsurvivors. The results of routine coagulation parameters measured at baseline and on days 3, 5, and 8 and a comparison between survivors and nonsurvivors are shown in Table 3.

### 3.2. Plasma FN Concentration

**Figure 2:**
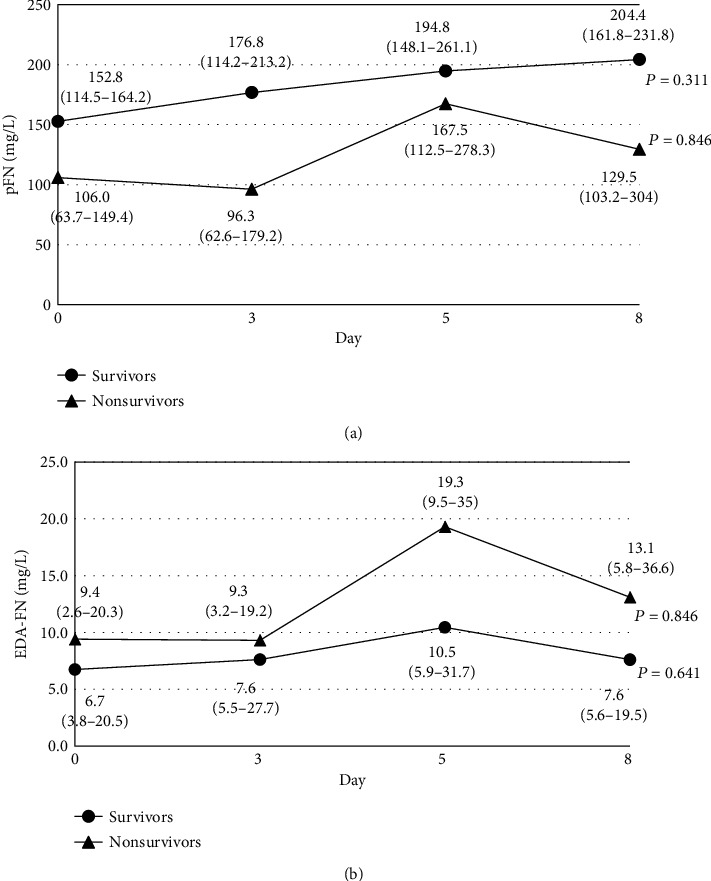
The time course of the changes in the pFN (a) and EDA-FN (b) levels in plasma of patients with sepsis. pFN and EDA-FN concentrations were determined by ELISA [25, 26]. The *P* values refer to the time course of the changes in pFN and EDA-FN levels in each group (Friedman repeated-measures ANOVA on ranks was applied to repeated measurements).

The pFN concentration of sepsis patients was significantly lower than in the control (133.0 mg/L vs. 231.2 mg/L) (*P* < 0.001). Analysis of the patient groups showed that the pFN concentration measured on day 0 (the first day in the ICU) was significantly lower in nonsurvivors (106.0 mg/L) compared to values recorded in survivors (152.8 mg/L) (*P* = 0.004). Additionally, patients with DIC had a lower pFN concentration than those without DIC (survivors: 138.8 mg/L vs. 154.2 mg/L; nonsurvivors: 82.8 mg/L vs. 108.3 mg/L, respectively).  Figure 2(a) shows the time course of the pFN level in consecutive days of ICU treatment in survivors and nonsurvivors. The median value of the pFN concentration increased continuously in subsequent days of observation in the survivors but not in nonsurvivors; however, these changes were not statistically significant in both sepsis groups.

### 3.3. EDA-FN Concentration

The median EDA-FN concentration measured on day 0 was slightly lower in survivors (6.7 mg/L) than in nonsurvivors (9.4 mg/L) and was significantly higher (*P* < 0.001) than in the control (1.4 mg/L). Additionally, the EDA-FN plasma concentration was markedly elevated in patients with DIC in the nonsurvivors (18.5 mg/L) and was low in survivors (4.8 mg/L). Changes in EDA-FN levels over time in sepsis patients are shown in Figure 2(b). The EDA-FN plasma concentration increased over time in both sepsis groups, but the highest increase was observed in the group of nonsurvivors between days 3 and 5 (1.4 times increase).

### 3.4. pFN and Isoform EDA-FN Levels in Relation to the Severity of Sepsis

Table 4 shows the baseline pFN and EDA-FN concentrations in relation to the severity of sepsis assessed using the APACHE II and SOFA clinical scales on admission to the ICU. Depending on the severity of a patient's condition, 2 ranges for the APACHE II scale and 2 for the SOFA scale were distinguished; higher scores on the scale indicate a more severe clinical condition of the patient. The pFN concentration in survivors was markedly higher than that in nonsurvivors in both APACHE II and SOFA ranges. The EDA-FN concentration was higher in nonsurvivors than in survivors, regardless of APACHEII score; however, the differences were not statistically significant. In addition, we observed that the highest EDA-FN concentration was in nonsurvivors with a SOFA score of more than 12 points; however, statistical significance was not achieved when comparing sepsis groups (*P* = 0.469) (Table 4).

### 3.5. FN Fragments

**Figure 3:**
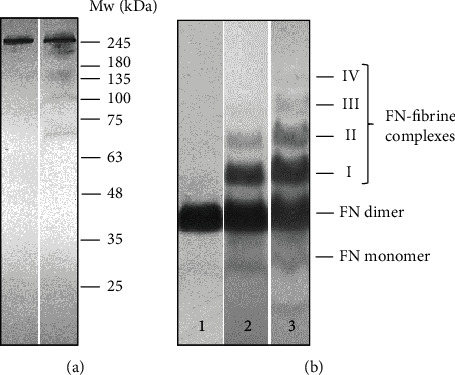
Representative immunopatterns of FN-fs (a) and FN-fibrin complexes (b) in plasma samples of patients with sepsis and control group. The plasma samples of sepsis patients and normal individuals were subjected to SDS (10%)-polyacrylamide gel (a) and SDS-agarose immunoblotting under nonreducing conditions (b). For details, see Materials and Method. The molecular weight of the FN band (a) was estimated using protein standard. Plasma samples (a) lane 1, control; lane 2, sepsis patient; (b) lanes 1-2, sepsis patients; lane 3, control. The molecular masses of the 750 to 1600 kDa plasma FN-fibrin complexes, 500 kDa FN dimer, and FN monomer are shown by arrows on the left.

Representative immunoblotting (Figure 3(a)) patterns of plasma FN samples revealed the presence of bands of high molecular masses (~250 kDa, ~220 kDa), corresponding to the polypeptides of FN isoforms and the fragmentation products (FN-fs) (~150, ~120, ~100, and~70 kDa). Fragmentation products of FN appeared in both sepsis groups but the frequency of occurrence was higher in nonsurvivors (68%) than in survivors (45%). In addition, the FN fragments with a molecular weight lower than 100 kDa were present in 46% of nonsurvivors patients and only in 36% of survivors.

### 3.6. Occurrence of FN-Fibrin Complexes

SDS-agarose immunoblotting revealed the presence bands corresponding to the FN dimer (500 kDa) and FN monomer (~250 kDa); in addition, the series of FN-fibrin bands with decreasing electrophoretic mobilities (Table 5, Figure 3(b)) and increasing molecular masses of 750, 1000, 1300, and 1600 kDa appeared. These bands were sequentially numbered as FN-fibrin I-IV complexes. The frequency of occurrence and relative amount of FN-fibrin complexes in the plasma of sepsis patients was higher in nonsurvivors than in survivors.

## 4. Discussion

The study showed that changes in the level of both forms of fibronectin, plasmatic (pFN) and cellular forms (EDA-FN), were associated with the severity of sepsis. The pFN concentration measured in sepsis patients was significantly lower than in the control samples from healthy adults, while the EDA-FN concentration was significantly higher than in the control. Comparing patient groups, it was shown that the pFN concentration in survivors was markedly higher than that in nonsurvivors in all APACHE II and SOFA ranges, and EDA-FN concentration was lower in survivors than in nonsurvivors. The initial pFN was associated with mortality in the univariate analysis and, together with the APACHE II score, in the multivariate analysis. In addition, we found that pFN levels in sepsis patients with DIC were lower compared to those without DIC and were particularly low in patients with DIC who died.

The main role of pFN is to participate in wound healing by interacting with fibrin during the coagulation process [5]. The reduced concentration of pFN observed in our study probably resulted from the excessive utilization of pFN during sepsis due to activation of the coagulation cascade. A low pFN level associated with DIC could have been a result of the depletion of plasma clotting components, often seen in DIC [29]. Comparing patient groups with similar severity of sepsis assessed using APACHE and SOFA scores, we observed that the pFN level was significantly higher in survivors than in nonsurvivors (Table 4). This indicates that higher pFN levels were a good prognostic marker for critically ill sepsis patients with a high result on the APACHE and SOFA scales. The univariate logistic regression showed that the initial pFN, APACHE II, SOFA, and PCT are independent predictors of mortality (Table 2), while the initial EDA-FN was not significant as a mortality predictor.

We found that EDA-FN level was significantly elevated in both sepsis groups; this observation was consistent with previously published results indicating a rapid increase in EDA-FN in pathological conditions [30–32]. Cellular forms of FN are produced by different types of cells (e.g., fibroblasts, smooth muscle cells, endothelial cells, platelets, and monocytes) [5], and in the blood of healthy people, the FN isoform with the EDA domain is absent or present at very low levels [5]. Previous research showed that the average concentrations of EDA-FN in blood samples of patients with sepsis were significantly higher than in healthy people [33], and the results of our study support this observation. The increased EDA-FN level in the blood of patients with sepsis is most likely associated with the activation of acute inflammation, severe injury, and coagulation abnormalities. Interestingly, the highest baseline concentration of EDA-FN was in the group of nonsurvivors with severe organ failure indicated by a high SOFA score.

Our study indicated the presence of FN-fibrin complexes with molecular masses from 750 to 1900 kDa in plasma samples of sepsis patients. These FN forms were evidently associated with the severity of sepsis, because they were absent in the control group. In our previous studies, FN-fibrin complexes with a molecular weight greater than 750 kDa were observed in the plasma of patients with various inflammatory disorders [28, 32]. Activation of the coagulation system and the conversion of fibrinogen to fibrin can lead to the formation of complexes with fibronectin. Therefore, the occurrence of FN-fibrin complexes in the plasma of patients suffering from sepsis might reflect the response of the organism to interconnected processes such as inflammation, immunity, and coagulation. Sepsis is often associated with coagulation abnormalities, and the antithrombin system, the activated protein C system, and the tissue factor pathway inhibitor (TFPI) are heavily disorganized [34].

## 5. Conclusions

Our data showed that the fibronectin plasma level was associated with the severity of sepsis. We observed that EDA-FN levels were significantly elevated, and pFN levels were significantly lower in the blood of patients with sepsis, and it appears that lower levels of pFN are associated with higher mortality. Our results show that the level of pFN in the context of the APACHE and SOFA scores may be an additional prognostic indicator in sepsis. However, to fully understand the role of fibronectin in sepsis, further research is needed; additional studies with large sample sizes should particularly help to characterise the role of FNs in the deterioration of vital organ function in sepsis.

## Figures and Tables

**Table 1:** Baseline characteristics of patients.

	Survivors	Nonsurvivors	Control	*P* value
	*n* = 35	*n* = 36	*n* = 17	
APACHE-II score	19.0 (13.0-25.0)	26.0 (20.0-32.0)		<0.001^a^
SOFA score	9.0 (6.0-11.0)	13.0 (10.0-15.0)		<0.001^a^
Procalcitonin (ng/L)	2.9 (0.5-12.9)	13.1 (2.8-33.0)		0.009^a^
C-reactive protein (mg/L)	169.0 (87.8-302.5)	178.6 (98.9-283.1)	1.2 (0.4-1.9)	1.00^a^
				<0.001^b,c^
White blood cells (10^3^/*μ*L)	15.0 (11.0-21.8)	15.1 (10.7-21.2)		0.875^a^
Haemoglobin (g/dL)	10.2 (8.2-11.6)	9.5 (8.6-10.7)		0.945^a^
Fibrinogen (g/L)	5.2 (4.1-6.5)	5.2 (2.9-5.7)		0.279^a^
pFN (mg/L)	152.8 (114.5-164.2)	106.0 (63.7-149.4)	231.2 (191.2-273.2)	0.004^a^
				<0.001^b,c^
EDA-FN (mg/L)	6.7 (3.8-20.5)	9.4 (2.6-20.3)	1.4 (0.9-1.6)	1.00^a^
				<0.001^b,c^
ICU free days to day 28	18 (9–21)	20 (9–24)		0.103^a^

APACHE II: Acute Physiology and Chronic Health Evaluation II; SOFA: Sequential Organ Failure Assessment; pFN: plasma fibronectin; EDA-FN: extra domain A fibronectin; ICU: intensive care unit. The comparison of continuous variables between two independent groups (nonsurvivors vs. survivors) was performed using the Mann-Whitney *U* test. The comparison of continuous variables across three groups (nonsurvivors, survivors, control) was performed using the Kruskal-Wallis test, and a post hoc was used to compare subgroups. The *P* value represents statistically significant differences calculated between the following groups: ^a^nonsurvivors vs. survivors, ^b^nonsurvivors vs. control, ^c^survivors vs. control.

**Table 2:** Univariate and multivariate analysis of possible risk factors for mortality.

Parameter	Univariate, OR (95% CI)	*P* value	Multivariate, OR (95% CI)	*P* value
pFN	0.992 (0.984-1.000)	0.046	0.985 (0.974-0.997)	0.010
APACHEII	1.136 (1.049-1.229)	0.001	1.17 (1.065-1.286)	0.001
SOFA	1.400 (1.145-1.713)	0.001		
PCT	1.015 (0.999-1.032)	0.011		

OR: odds ratio; CI: confidence interval; SOFA: Sequential Organ Failure Assessment; APACHE: Acute Physiology and Chronic Health Evaluation; PCT: procalcitonin.

**Table 3:** The routine parameters of the coagulation system.

	Day	Survivors	Nonsurvivors	*P* value^∗∗^
Platelets	1	205 (156-330)	131 (57-224)	0.006
Ref. range: 140-440 (10^3^/*μ*L)	3	190 (128-301)	101 (43-168)	<0.001
	5	203 (107-280)	114 (32-177)	<0.001
	8	205 (114-247)	140 (87-177)	0.125
	*P* value^∗^	0.017	0.001	
INR	1	1.2 (1.1-1.4)	1.4 (1.3-1.7)	<0.001
Ref. range: 0.9-1.3	3	1.1 (1.1-1.2)	1.4 (1.2-1.9)	<0.001
	5	1.1 (1.0-1.1)	1.2 (1.1-1.4)	0.005
	8	1.1 (1.0-1.2)	1.2 (1.1-1.3)	0.034
	*P* value^∗^	<0.001	<0.001	
D-dimers	1	4.5 (2.1–6.2)	6.1 (4.0–17.6)	0.039
Ref. range: 0.0-0.5 *μ*g/mL	3	3.7 (2.9–6.0)	5.0 (1.9–21.8)	0.367
	5	4.2 (2.3–11.0)	6.1 (3.1–25.0)	0.536
	8	8.6 (5.1–14.5)	3.8 (2.5–5.2)	0.267
	*P* value^∗^	0.111	0.614	

^∗^Friedman ANOVA test, ^∗∗^Mann-Whitney *U* test. PLT: platelet count; INR: international normalized ratio.

**Table 4:** Baseline pFN and EDA-FN concentrations in the plasma of patients with sepsis in relation to the APACHE II and SOFA score calculated on admission to the ICU.

	pFN (mg/L)		*P* value	EDA-FN (mg/L)		*P* value
	Survivors	Nonsurvivors		Survivors	Nonsurvivors	
APACHE II ≤ 20	137.1 (113.4-156.4)	106.8 (66.4-122.0)	0.031	6.7 (4.0-29.5)	11.0 (6.0-23.6)	0.841
APACHE II > 20	157.0 (153.5-196.7)	99.8 (62.7-159.4)	0.029	4.2 (3.8-12.6)	5.8 (2.5-23.1)	0.727
SOFA ≤ 12	152.3 (113.4-168.2)	93.1 (56.7-133.6)	0.002	7.7 (4.0-29.5)	7.6 (2.9-21.2)	0.584
SOFA > 12	138.8 (119.3-154.6)	109.9 (62.7-166.6)	0.410	3.9 (3.5-12.2)	12.6 (2.8-25.6)	0.469

The APACHE II scale: in survivors, 57% of patients had APACHE II ≤20, and 43% had APACHE II >20; in nonsurvivors, the proportions were reversed, i.e., 28% of patients had APACHE II ≤20, and 72% had APACHE II >20 (*P* = 0.023). The SOFA scale: in survivors, 87% of patients had SOFA ≤12, and 13% had SOFA >12; in nonsurvivors, the proportions were reversed, i.e., 38% of patients had SOFA ≤12, and 62% had SOFA >12 (*P* < 0.001).

**Table 5:** The appearance of FN monomers, FN dimers, and FN-fibrin complexes in the plasma of patients with sepsis determined on the basis of immunoblotting after SDS-AGE patterns.

		Survivors	Nonsurvivors	Control	*P* value
FN monomer	Occurrence (%0	80	80	88	0.745
	Relative amount (median (IQR))	4.4 (3.5-6.7)	5.6 (3.8-7.0)	5.7 (3.8-6.6)	0.940^1^
					1.000^2^
					1.000^3^
FN dimer	Occurrence (%)	100	100	100	—
	Relative amount (median (IQR))	63.0 (52.5-73.8)	56.8 (46.8-64.4)	92.7 (92.4-95.9)	0.342^1^
					<0.001^2^
					<0.00^3^1
FN-fibrin complexes					
I	Occurrence (%)	100	100	18	<0.001
	Relative amount (median (IQR))	28.5 (16.0-31.8)	29.0 (23.5-32.7)	3.8 (3.7-8.7)	0.766^1^
					0.030^2^
					0.007^3^
II	Occurrence (%)	89	94	6	<0.001
	Relative amount (median (IQR))	5.3 (2.5-11.3)	7.4 (3.8-12.9)	1.8 (1.8-1.9)	0.656^1^
					0.554^2^
					0.310^3^
III	Occurrence (%)	46	67	0	<0.001
	Relative amount (median (IQR))	3.2 (1.8-6.0)	3.0 (1.6-6.3)	Not detected	1.000
IV	Occurrence (%)	20	33	0	0.021
	Relative amount (median (IQR))	1.1 (0.7-1.7)	1.3 (0.7-2.0)	Not detected	1.000

*P* value represents difference between subgroups: ^1^*P*—survivors vs. nonsurvivors; ^2^*P*—survivors vs. control; ^3^*P*—nonsurvivors vs. control. The comparison of continuous variables between two independent groups (nonsurvivors vs. survivors) was performed using the Mann-Whitney *U* test. The comparison of continuous variables across three groups (nonsurvivors, survivors, and control) was performed using the Kruskal-Wallis test, and a post hoc was used to compare subgroups. Plasma FN forms were revealed by SDS-agarose immunoblotting (see Figure 3(b)). Occurrence is the ratio of the number of samples containing the FN form to the total number of samples. The relative amount of the FN band is the percentage of the total number of pixels found in the electrophoresis path.

## Data Availability

The data that support the findings of this study are available on request from the corresponding author Anna Lemańska-Perek. The data have not been made publicly available because they contain information that could compromise the privacy of the study participants.

## Supplementary Materials

Supplementary Materials Table S1: the development of inflammatory response monitored by the concentration of C-reactive protein, procalcitonin, and WBC level.

## References

[1] Singer M., Deutschman C. S., Seymour C. W. (2016). The third international consensus definitions for sepsis and septic shock (Sepsis-3). *Journal of the American Medical Association*.

[2] Sakr Y., Jaschinski U., Wittebole X. (2018). Sepsis in intensive care unit patients: worldwide data from the intensive care over nations audit. *Open Forum Infectious Diseases*.

[3] Schouten M., Wiersing W. J., Levi M., van der Poll M. (2008). Inflammation, endothelium, and coagulation in sepsis. *Journal of Leukocyte Biology*.

[4] Adamik B., Goździk W., Jakubczyk D., Wełna M., Kübler A. (2017). Coagulation abnormalities identified by thromboelastometry in patients with severe sepsis: the relationship to endotoxemia and mortality. *Blood Coagulation & Fibrinolysis*.

[5] To W. S., Midwood K. S. (2011). Plasma and cellular fibronectin: distinct and independent functions during tissue repair. *Fibrogenesis & Tissue Repair*.

[6] Pankov R., Yamada K. M. (2002). Fibronectin at a glance. *Journal of Cell Science*.

[7] White E. S., Baralle F. E., Muro A. F. (2008). New insights into form and function of fibronectin splice variants. *The Journal of Pathology*.

[8] Rohwedder I., Montanez E., Beckmann K. (2012). Plasma fibronectin deficiency impedes atherosclerosis progression and fibrous cap formation. *EMBO Molecular Medicine*.

[9] Zhang Y., Zhou X., Krepinsky J. C., Wang C., Segbo J., Zheng F. (2006). Association study between fibronectin and coronary heart disease. *Clinical Chemistry and Laboratory Medicine*.

[10] Muro A. F., Moretti F. A., Moore B. B. (2008). An essential role for fibronectin extra type III domain A in pulmonary fibrosis. *American Journal of Respiratory and Critical Care Medicine*.

[11] Jarnagin W. R., Rockey D. C., Koteliansky V. E., Wang S. S., Bissell D. M. (1994). Expression of variant fibronectins in wound healing: cellular source and biological activity of the EIIIA segment in rat hepatic fibrogenesis. *The Journal of Cell Biology*.

[12] Cappellari G. G., Barazzoni R., Cattin L., Muro A. F., Zanetti M. (2016). Lack of fibronectin extra domain A alternative splicing exacerbates endothelial dysfunction in diabetes. *Scientific Reports*.

[13] Rybak J. N., Roesli C., Kaspar M., Vila A., Neri D. (2007). The extra-domain A of fibronectin is a vascular marker of solid tumors and metastases. *Cancer Research*.

[14] Sakai T., Johnson K. J., Murozono M. (2001). Plasma fibronectin support neuronal survival and reduces brain injury following transient focal cerebral ischemia but is not essential for skin-wound healing and hemostasis. *Nature Medicine*.

[15] Shinji H., Yosizawa Y., Tajima A. (2011). Role of fibronectin-binding proteins A and B in vitro cellular infections and in vivo septic infections by staphylococcus aureus. *Infection and Immunity*.

[16] Schröder A., Schröder B., Roppenser B. (2006). Staphylococcus aureus fibronectin binding protein-A induces motile attachment sites and complex actin remodelling in living endothelial cells. *Molecular Biology of the Cell*.

[17] Martín G. R., Prieto J. P., Veiga de Cabo J. (2004). Plasma fibronectin as a marker of sepsis. *International Journal of Infectious Diseases*.

[18] Reichsoellner M., Raggam R. B., Wagner J., Krause R., Hoenigl M. (2014). Clinical evaluation of multiple inflammation biomarkers for diagnosis and prognosis for patients with systemic inflammatory response syndrome. *Journal of Clinical Microbiology*.

[19] Knaus W. A., Draper E. A., Wagner D. P., Zimmerman J. E. (1985). APACHE II. *Critical Care Medicine*.

[20] Vincent J. -L., Moreno R., Takala J. (1996). The SOFA (Sepsis-related Organ Failure Assessment) score to describe organ dysfunction/failure. *Intensive Care Medicine*.

[21] Rhodes A., Evans L. E., Alhazzani W. (2017). Surviving sepsis campaign: international guidelines for management of sepsis and septic shock: 2016. *Intensive Care Medicine*.

[22] Toh C. H., Hoots W. K., SSC on Disseminated Intravascular Coagulation of the ISTH (2007). The scoring system of the Scientific and Standardisation Committee on Disseminated Intravascular Coagulation of the International Society on Thrombosis and Haemostasis: a 5 year overview. *Journal of Thrombosis and Haemostasis*.

[23] Young P. J., Weatherall M., Saxena M. K. (2013). Statistical analysis plan for the HEAT trial: a multicentre randomised placebo-controlled trial of intravenous paracetamol in intensive care unit patients with fever and infection. *Critical Care and Resuscitation*.

[24] Young P., Hodgson C., Dulhunty J. (2012). End points for phase II trials in intensive care: recommendations from the Australian and New Zealand Clinical Trials Group consensus panel meeting. *Critical Care and Resuscitation*.

[25] Lemańska-Perek A., Pupek M., Polańska B., Kątnik-Prastowska I. (2013). Alterations in molecular status of plasma fibronectin associated with aging of normal human individuals. *Clinical Biochemistry*.

[26] Ziffels B., Ospel J., Grün K. (2016). Detection of soluble ED-A+ fibronectin and evaluation as novel serum biomarker for cardiac tissue remodeling. *Disease Markers*.

[27] Pupek M., Krzyżanowska-Goląb D., Dyła T., Lemańska-Perek A., Jankowska R., Kątnik-Prastowska I. (2009). Presence of high-molecular-weight forms and domain alterations of fibronectin in pleural effusion of patients with lung cancer. *Clinical Biochemistry*.

[28] Krzyżanowska-Gołąb D., Lemańska-Perek A., Pupek M. (2014). Identification of soluble supramolecular FN-fibrin complexes in human plasma. *Journal of Immunoassay & Immunochemistry*.

[29] Zeerleder S., Hack C. E., Wuillemin W. A. (2005). Disseminated intravascular coagulation in sepsis. *Chest*.

[30] van Keulen J. K., de Kleijn D. P., Nijhuis M. M. O. (2007). Levels of extra domain A containing fibronectin in human atherosclerotic plaques are associated with a stable plaque phenotype. *Atherosclerosis*.

[31] von Au A., Vasel M., Kraft S. (2013). Circulating fibronectin controls tumor growth. *Neoplasia*.

[32] Lemańska-Perek A., Krzyżanowska-Gołąb D., Pupek M., Klimeczek P., Witkiewicz W., Kątnik-Prastowska I. (2016). Analysis of soluble molecular fibronectin-fibrin complexes and EDA-fibronectin concentration in plasma of patients with atherosclerosis. *Inflammation*.

[33] Satoi S., Kitade H., Hiramatsu Y. (2000). Increased extra domain-A containing fibronectin and hepatic dysfunction during septic response: an in vivo and in vitro study. *Shock*.

[34] Levi M., van der Poll T. (2017). Coagulation and sepsis. *Thrombosis Research*.

