# Supplementary material for: Plasma and Cellular Forms of Fibronectin as Prognostic Markers in Sepsis
Source: Mediators Inflamm. 2020 Aug 1;2020:8364247. doi: 10.1155/2020/8364247 (PMC7416265; doi:10.1155/2020/8364247)
Supplement: Supplementary Materials — Table S1: the development of inflammatory response monitored by the concentration of C-reactive protein, procalcitonin, and WBC level. [file 8364247.f1.docx]

Table s1. The development of inflammatory response monitored by the concentration of C- reactive protein, procalcitonin, and WBC level.

|  | **Day** | **Survivors** | **Non-survivors** | ***P* value**** |
| --- | --- | --- | --- | --- |
| **C- reactive protein** | **1** | 169 (87-302) | 178 (98-283) | 0.956 |
| Ref. range: | **3** | 178 (132-262) | 180 (104-180) | 0.622 |
| 0.0 - 5.0 mg/L | **5** | 170 (112-246) | 105 (74-173) | 0.074 |
|  | **8** | 103 (60-173) | 167 (115-215) | 0.180 |
|  | ***P* value*** | <0.001^a^ | 0.371 |  |
| **Procalcitonin** | **1** | 3.0 (0.5-13.0) | 13.2 (2.8-33.0) | 0.009 |
| Ref. range: | **3** | 4.1 (0.8-22.4) | 10.2 (3.5-25.4) | 0.208 |
| <0.05 ng/mL | **5** | 1.6 (0.5-11.1) | 6.9 (1.4-11.2) | 0.180 |
|  | **8** | 1.1 (0.4-8.6) | 3.5 (0.9-6.1) | 0.288 |
|  | ***P* value*** | <0.001^b^ | <0.001^c^ |  |
| **WBC** | **1** | 15 (11-22) | 15 (11-21) | 0.868 |
| Ref. range: | **3** | 13 (10-17) | 13 (8-24) | 0.522 |
| 4 – 10^3^/µL | **5** | 13 (10-16) | 13 (9-31) | 1.000 |
|  | **8** | 12 (10-16) | 12 (11-26) | 0.110 |
|  | ***P* value*** | <0.001^d^ | 0.004^e^ |  |

*Friedman ANOVA test, **Mann-Whiney U test.

The Friedman repeated-measures ANOVA on ranks with the post-hoc test indicates statistically significant differences:

^a^ C- reactive protein level recorded between days 1 and 8, 3 and 8, 5 and 8.

^b^ Procalcitonin level recorded between days 1 and 5, 1 and 8, 2 and 5, 2 and 8.

^c^ Procalcitonin level recorded between days 1 and 8.

^d^ Procalcitonin level recorded between days 1 and 8.

^e^ Procalcitonin level recorded between days 2 and 8.
